# Migration of an Interactive Global Health Conference to a Virtual Platform: Engaging Learners During the Pandemic

**DOI:** 10.7759/cureus.25601

**Published:** 2022-06-02

**Authors:** Kacper Kubiszewski, Aaishwariya Gulani, Kelly Sutter, Bernard Sarmiento, Yasmine S Ghattas, Reanne Mathai, Judith S Simms-Cendan

**Affiliations:** 1 Medical School, University of Central Florida College of Medicine, Orlando, USA; 2 Obstetrics and Gynecology, University of Miami Miller School of Medicine, Miami, USA

**Keywords:** covid-19, virtual conference, student-led, service learning, medical education, conference, global health

## Abstract

The COVID-19 pandemic has halted many large gatherings, and research conferences are no exception. Large conferences, once attended in-person, have primarily switched to a virtual format, utilizing online platforms. Every January, Medical Students Providing Across Continents (MedPACt), the University of Central Florida College of Medicine’s global health interest group, hosts a student-run Global Health Conference that features a keynote speaker, discussion panel, and research presentations, and workshops for participants to engage in. Though planning this event is always challenging, organizing the 2021 conference was particularly strenuous as accommodations had to be made to optimize the conference to fit a never-attempted virtual format.

This drastic shift warrants further investigation into the efficacy and audience engagement of the virtual format. Using a post-conference survey with specific questions geared towards each component of the conference along with registration data, the virtual conference in 2021 was compared to the in-person conference in 2020. This study found that the virtual format was comparably efficacious in creating relevant and global health-oriented programming for the 2020 in-person conference. Additionally, the 2021 virtual conference received more registrants and cost less to plan, meaning the virtual model is a cost-effective way to deliver quality conference content.

## Introduction

Medical Students Providing Across Continents (MedPACt) is the University of Central Florida College of Medicine’s global health interest group. MedPACt hosts a Global Health Conference (GHC) that brings in a keynote speaker, discussion panel, research presentations, and workshops every year. The conference’s theme changes every year, with past topics including Refugee Health, Maternal and Infant Health, and Climate Change and Global Health. In 2020, the conference theme was Arts in Global Medicine. During the year leading up to the conference, student-led committees are formed to plan the conference and tailor the activities and speakers toward the year’s theme. 

Though planning this event is always challenging, 2021 was particularly strenuous as accommodations had to be made to optimize the conference to fit a never-before-attempted virtual format. Naturally, the theme selected for the 2021 GHC was Pandemics and Global Health, and the conference explored the global impacts of the COVID-19 crisis. Students chose to host the conference with a modified schedule using the Zoom Webinar platform, keeping the main elements of the conference (a keynote speaker, discussion panels, research, and workshops) intact. Zoom is an online video conferencing platform. It was selected over similar products such as Cisco Webex, Google Hangouts Meet, and GoToMeeting, primarily due to its ubiquity in medical education.

Although this was the first time the Global Health Conference was hosted in this virtual format, the student organizers remained optimistic regarding conference outcomes. Before the pandemic, video conferencing software established itself as a legitimate means of communicating and connecting with others. During the pandemic, one review maintained that positive technology use could improve personal and family life by facilitating scheduling flexibility and enhancing connection in the lives of those working or studying from home [1]. In the realm of medical student education, this was also at play as institutions mitigated the risks of COVID-19. In-person events needed to be rethought and re-organized into online platforms where possible, and this change occurred at undergraduate and graduated medical education levels [2]. Virtual orientations and educational sessions are routinely being scheduled to replace the need for face-to-face interaction [3]. For this reason, medical student perspectives were essential to keep in mind while planning a virtual conference. One study reveals that some students are happy with “online med school,” as conducting sessions during Zoom gives them a better opportunity to multi-task and have greater control of their time [4].

It was also crucial for organizers to garner feedback from other conferences that had to switch to a virtual format. Although the literature on this subject remains slim, some notable success stories have been. The Cochrane Skin conference had to quickly switch to a virtual platform in May of 2020 during the height of the pandemic, and its organizers shared some of the benefits and barriers related to virtual conferences [5]. They noted the advantages of decreased cost for organizers and increased accessibility for participants, as no travel is required. Another study corroborates the benefit to attendees by examining a variety of scientific conferences globally, explicitly citing an increase in participation and international representation as barriers such as cost and obtaining visas were eliminated. One particularly remarkable case is the American Society of Nutrition’s virtual conference, which increased its attendance from 3,157 attendees representing 59 countries in 2019 to over 30,000 representing 164 countries in one year [6].

Student organizers must be aware that these studies have recommendations and potential shortcomings. In its reflection, the Cochrane Skin conference organizers noted the barrier associated with reduced social and networking opportunities due to the virtual nature of its conference [5]. To attempt to help with this issue, GHC organizers hosted many events throughout the conference day that featured breakout rooms to allow more opportunities to engage with attendees, faculty, and guest speakers. Other studies have recommended testing the technology prior to the conference day, which was accomplished by a “dry-run” the week leading up to the virtual conference [7]. Organizers logged in through the Zoom Webinar platform and walked the GHC Board and student volunteers through the various presentations and stages of the conference, ensuring all transitions and cues were smooth.

However, the biggest concern going into the conference was attendee engagement and attentiveness. Known as “Zoom fatigue,” many have experienced this phenomenon as they transition to working from home in remote meetings. This includes medical students. Many note that they sometimes feel emotionally or mentally drained by back-to-back Zoom calls [6]. This was confirmed by another study, as although it maintained that virtual medical education could be effective, one of the downsides of virtual learning was the diminished engagement of students [8]. One study attempted to explain the cause of the Zoom fatigue phenomenon as a form of “nonverbal overload,” pointing out that there are several contributing factors to the phenomenon, such as excessive screen time, reduced physical mobility during videoconferencing, increased cognitive load, and the constant unnatural viewing of one’s image [9]. The GHC team implemented several changes during planning to mitigate this challenge, which included the use of breakout rooms during interactive workshops. In addition to the built-in Zoom chat system, Zoom breakout rooms allowed participants to join smaller virtual rooms and engage in discussions on specific topics with moderators and other attendees. Also, workshop events were planned to be interactive with educational games, simulated role-playing, and discussion groups. Lastly, the conference duration was shortened to five hours, and two five-minute breaks and one ten-minute break were implemented between events to help mitigate fatigue.

In order to assess and adapt for future years, the conference team surveyed participants after both the 2020 and 2021 conferences. An anonymous post-conference survey was sent out to assess the efficacy of the conference in teaching participants about global health and providing relevant insights into their professions. In 2021, this survey was adapted to ask the same questions and was sent out after the conference’s conclusion. This study aims to assess the efficacy of the virtual conference compared to the in-person conference in garnering interest in registration and providing content relevant to global health and participant profession.

## Materials and methods

The University approved the conduction of this study by the Central Florida Institutional Review Board under the title: “Investigation of Impact on Global Health Engagement in Virtual vs. In-Person Conference.” It was designated as STUDY00002569. 

Surveys were sent to participants via email after both conferences. The Qualtrics platform was utilized to create the survey and collect the results. Conference registration data was also obtained and analyzed. In 2020, organizers utilized Google Forms, while in 2021, a combination of WordPress and Stripe was used. The number of registrants by year and the state of the registrant’s affiliate institution were collected. In 2020, 230 surveys were sent out (equaling the total number of registrants) on January 18, 2020. Fifty-five individuals responded, yielding a response rate of 23.9%. In 2021, 351 surveys were sent out (equaling the total number of registrants) on January 16, 2021. Sixty-eight individuals responded, yielding a response rate of 19.4%. 

The Qualtrics survey contained a multiple-choice, Likert scale survey that asked participants to rate their extent of agreement with a given statement. There were five answer choices arranged in the following way: “strongly agree,” “agree,” “neither agree nor disagree,” “disagree,” and “strongly disagree.” For each question, the percentage of participants who chose a given answer was noted and separated by year. Seven Likert-scale questions were used in both years. Due to the categorical nature of this data, these results were then analyzed using a chi-squared test for independence within STATA. This was to determine whether there was a significant difference in the frequency of answer choices between the in-person 2020 and virtual 2021 conferences.

Two-sample t-testing was also performed within STATA to compare the mean Likert scores between the two groups. The qualitative categories of “strongly disagree,” “disagree,” “neither agree nor disagree,” “agree,” and “strongly agree” were converted into the values of 1, 2, 3, 4, and 5, respectively, for each of the seven questions. These values were summed and then divided by the number of questions, or seven, to form an overall satisfaction score for every response. The mean and standard deviation were then calculated for 2020 and 2021 respondent groups and used in t-testing.

## Results

Two hundred and thirty individuals registered for the 2020 GHC, while 351 registered in 2021, yielding a 53% increase in attendance (Table 1). In 2020, the data from registrants that designated an affiliate institution revealed two registrants affiliated with a non-Florida program, each from a different state (Table 1). In 2021, 29 registrants were affiliated with a non-Florida program, encompassing eight different non-Florida states (Table 1).

**Table 1:** 2020 and 2021 Global Health Conference In-State and Out-of-State Registration

	In-State	Out-of-State	Not Available*	Total
2020	225 (97.82%)	2 (0.87%)	3 (1.30%)	230
2021	308 (87.75%)	29 (8.27%)	14 (3.99%)	351
*The number of participants that did not disclose their affiliate institution				

Across both years, overall survey respondent satisfaction was high, which was evident by the high percentage of “strongly agree” and “agree” results across many of the survey questions. Significantly more respondents felt that the keynote speaker was relevant to their profession in 2021 (p=0.02, Table 2). No significant difference was noted in responses to the keynote speaker session providing insight into global health (p=0.10, Table 2). Significantly more individuals reported learning more about global health in 2021 through the panelist and roundtable sessions (p=0.02, Table 2, p=0.01, Table 2). Additionally, significantly more individuals in 2021 felt that the poster presentation was helpful in their profession (p=0.000, Table 2). No significant difference was observed in participant ratings of either the workshops (p=0.20, Table 2) or the overall number of knowledge/skills learned from the conference (p=0.20, Table 2). Nonetheless, it is interesting to point out that a higher relative proportion of individuals in 2021 reported “strongly agree” in all questions analyzed compared to 2020 (Table 2). 

**Table 2:** Likert Scale Survey Responses from the 2020 and 2021 Global Health Conferences

Survey Questions		
To what extent do you agree with the following statement: I found the keynote talk relevant to my profession (X^2^=10.1; p=0.02)		
	2020	2021
Strongly agree	44.40%	69.10%
Agree	37.00%	26.50%
Neither agree nor disagree	16.70%	4.40%
Disagree	1.90%	0%
Strongly disagree	0.00%	0.00%
Likert Scale Mean	4.24 (SD=0.80)	4.65 (SD=0.57)
To what extent do you agree with the following statement: I gained more insight in Global Medicine by our Keynote Speaker (X^2^=6.2; p=0.10)		
	2020	2021
Strongly agree	53.70%	66.20%
Agree	29.60%	27.90%
Neither agree nor disagree	16.70%	4.40%
Disagree	0%	1.50%
Strongly disagree	0.00%	0.00%
Likert Scale Mean	4.37 (SD=0.76)	4.59 (SD=0.65)
To what extent do you agree with the following statement: I was able to learn more about global health from the Panelist session (X^2^=9.9; p=0.02)		
	2020	2021
Strongly agree	48.10%	72.10%
Agree	29.60%	22.10%
Neither agree nor disagree	20.40%	5.90%
Disagree	1.90%	0%
Strongly disagree	0.00%	0.00%
Likert Scale Mean	4.24 (SD=0.85)	4.66 (SD=0.59)
To what extent do you agree with the following statement: I was able to learn more about global health from the roundtable discussion (X^2^=10.9; p=0.01)		
	2020	2021
Strongly agree	33.30%	63.20%
Agree	35.20%	20.60%
Neither agree nor disagree	27.80%	14.70%
Disagree	3.70%	1.50%
Strongly disagree	0.00%	0.00%
Likert Scale Mean	3.98 (SD=0.88)	4.46 (SD=0.80)
To what extent do you agree with the following statement: The poster presentation was useful to my profession (X^2^=15.8; p=0.00)		
	2020	2021
Strongly agree	25.90%	45.60%
Agree	31.50%	30.90%
Neither agree nor disagree	42.60%	19.10%
Disagree	0%	4.40%
Strongly disagree	0%	0%
Likert Scale Mean	3.83 (SD=0.82)	4.37 (SD=0.69)
To what extent do you agree with the following statement: The workshops were beneficial (X^2^=3.3; p=0.20)		
	2020	2021
Strongly agree	50.00%	64.70%
Agree	44.40%	33.80%
Neither agree nor disagree	5.60%	1.50%
Disagree	0%	0%
Strongly disagree	0%	0%
Likert Scale Mean	4.45 (SD=0.61)	4.63 (SD=0.52)
To what extent do you agree with the following statement: Overall, I learned/gained a substantial amount of knowledge/skills from the conference (X^2^=3.2; p=0.20)		
	2020	2021
Strongly agree	41.80%	57.40%
Agree	50.90%	39.70%
Neither agree nor disagree	7.30%	2.90%
Disagree	0%	0%
Strongly disagree	0%	0%
Likert Scale Mean	4.35 (SD=0.62)	4.54 (SD=0.56)

Overall, the mean Likert score in 2021 was higher [4.56 (SD=0.45)] compared to the mean Likert score in 2020 [4.20 (SD=0.57)]. This represents a significant difference (p=0.0002) and increases respondent satisfaction with the 2021 virtual conference compared to the 2020 in-person conference. In terms of individual question items, “The Poster Presentation was useful to my profession” had the lowest mean scores in both the 2020 (3.83, SD=0.82) and 2021 (4.37, SD=0.69) groups, representing a potential target for improvement. However, this question item had the most significant score increase from 2020 to 2021 (+0.53). In 2020 and 2021, respondents were most satisfied with the keynote speaker and workshop sessions. 

**Figure 1:**
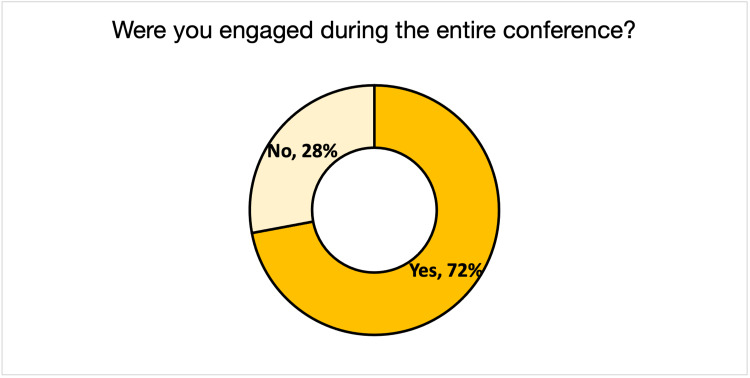
Audience Engagement During the 2021 Global Health Conference

**Figure 2:**
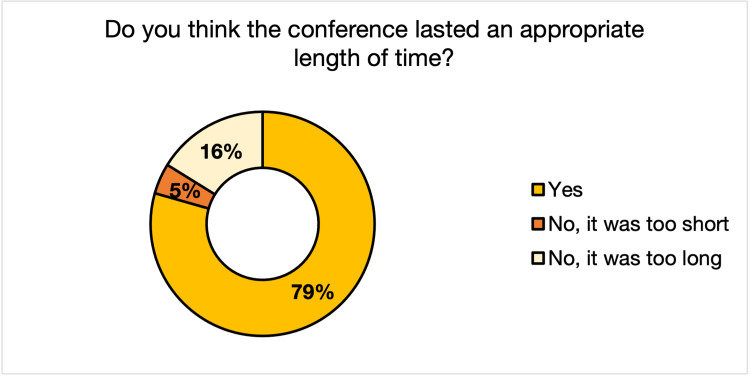
Audience Perception of 2021 Global Health Conference Duration

**Figure 3:**
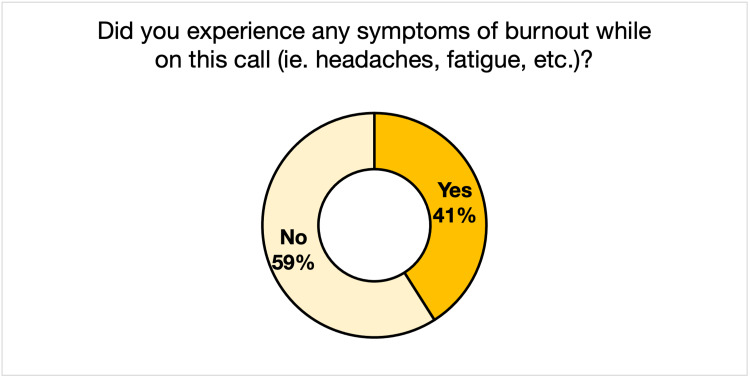
Audience Perception of Burnout Symptoms During the 2021 Global Health Conference

Regarding the standalone questions, 72% of the respondents stated they remained engaged throughout the virtual conference (Figure 1). Seventy-nine percent thought that the conference lasted an appropriate amount of time, while 16% thought it was too long, and 5% thought it was too short (Figure 2). Fifty-nine percent of respondents stated that they did not experience any symptoms of burnout during the conference, while 41% did (Figure 3).

## Discussion

Registration increased 53% from 2020 to 2021, with a substantial increase in out-of-state registrants. This phenomenon is not unique to the GHC, as one study on five international conferences showed increased attendance from lower and middle-income countries [10]. Across all aspects of the conference (keynote speaker, discussion panel, roundtable discussion, poster presentations, and workshops), respondents rated the 2021 conference to be more effective or comparably practical than the 2020 conference in terms of the relevance of its programming to global health and participant professions. The mean Likert scores for 2020 and 2021 revealed that respondents were more satisfied with the virtual GHC than the 2020 in-person event. However, the two conferences were thematically different, making this an imperfect conclusion. Though many did experience symptoms of burnout during the call (41%), the majority of the 2021 GHC respondents (72%) felt that they remained engaged throughout the conference, and only a minority (16%) deemed the conference to be too long.

The 2021 virtual conference was also logistically beneficial when considering the conference’s planning. When planning an in-person conference, items such as catering food, hotel accommodations, and travel fees for speakers, decorations, print media, and other supplies must be considered by organizers to ensure the conference does not exceed its budget. These items, plus tasks like room assignments and volunteer coordination, also mean more logistical planning and “moving parts” during the conference day, making planning an in-person conference more difficult for organizers. In terms of the total cost, the 2020 conference cost approximately $6,900; in contrast, the 2021 conference cost approximately $2,700. The 2021 budget went towards purchases such as speaker fees and a license for a Zoom Webinar. This seems to be confirmed by a strengths, weaknesses, opportunities, and threats (SWOT) analysis performed on virtual conferences. Researchers note that a significant strength of the virtual format is the shorter and more manageable planning phase for organizers and easier accessibility for registrants [11].

The limitations of this study should also be noted. Though the registration data was helpful, it should be noted that registration did not necessarily equal participation, and the number of conference attendees were not tracked. It is also possible that individuals that did not attend the conference filled out the survey. Furthermore, the change in theme from 2020 to 2021 is a confounding variable “Arts in Medicine” and “Global Pandemics” are vastly different subjects. The COVID-19 pandemic was such a universal experience that it impacted almost everyone; perhaps participants felt like they resonated more with the theme, which is why interest was higher, and participants rated the 2021 conference to be more relevant. Additionally, given that many attendees were in the healthcare field, COVID-19 likely had a stronger clinical connection than “Arts in Medicine,” meaning that more participants may have felt that COVID-19 as a topic was inherently more relevant to global health and their profession.

## Conclusions

This study demonstrates that the 2021 virtual conference was more effective than the 2020 in-person conference in garnering interest in registration and providing content relevant to global health and participant profession. The keynote speaker, discussion panel, roundtable discussion, research posters, and workshop sessions all received similar or improved ratings from 2020 to 2021, and the mean Likert score was higher in 2021. Participants also reported remaining engaged during the virtual conference, thereby mitigating the concerns of having an uninteresting conference due to an online format. The 2021 virtual conference received more registrants, including an uptick in out-of-state registrants. This, combined with the cost-effectiveness and logistical advantages of a virtual format, means that virtual conferences are a viable way to engage learners and expand audience pools.
